# Total arch replacement for nondissecting aortic arch aneurysms: Risk stratification and long-term outcomes

**DOI:** 10.1016/j.xjon.2026.101943

**Published:** 2026-06-29

**Authors:** Katsuhiro Yamanaka, Taishi Inoue, Daiki Kato, Yuta Nakaoka, Takuya Wada, Ryoichi Furutani, Kazunori Sakaguchi, Tasuku Okada, Jun Karaki, Hironaga Shiraki, Ryo Kawabata, Noriko Ohyama, Soichiro Henmi, Hiroaki Takahashi, Kenji Okada

**Affiliations:** Division of Cardiovascular Surgery, Department of Surgery, Kobe University Graduate School of Medicine, Kobe, Japan

**Keywords:** aortic arch aneurysm, total arch replacement, endovascular aortic repair, risk stratification

## Abstract

**Objectives:**

This study evaluated 25-year outcomes and established risk stratification in patients undergoing total arch replacement with a 4-branched graft for nondissecting aortic arch aneurysm.

**Methods:**

From October 1999 to December 2024, 554 patients were retrospectively analyzed. The primary end point was long-term overall survival. Secondary end points included 30-day mortality, hospital mortality, freedom from aorta-related death, and freedom from aortic events. Subgroup analyses stratified patients by age, renal function, their combination, and the number of risk factors for late mortality, and survival was compared across groups.

**Results:**

Thirty-day and hospital mortality were 1.0% and 3.6%, respectively. At 10 years, overall survival, freedom from aorta-related death, and freedom from aortic events were 51.8 ± 2.8%, 89.5 ± 1.9%, and 70.5 ± 2.8%, respectively. Patients aged ≥85 years had significantly worse survival than younger groups. Those with estimated glomerular filtration rate (eGFR) ≥45 mL/min/1.73 m^2^ showed significantly better survival than those with eGFR <45. Multivariable analysis identified age ≥85 years, eGFR <45, chronic obstructive pulmonary disease, history of cerebrovascular disease, and peripheral artery disease as independent preoperative risk factors for late mortality. Long-term survival decreased significantly as the number of risk factors increased (*P* < .0001).

**Conclusions:**

Total arch replacement provides durable long-term outcomes with excellent freedom from aorta-related death. Optimal treatment should be individualized using a patient-centered approach determined on the basis of preoperative risk factors.

**Figure:**
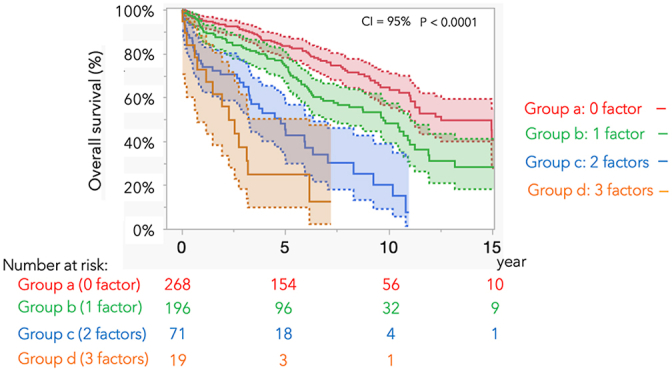
Survival decreases with increasing preoperative risk factors (0-≥3).

**Central Message**

Total arch replacement provides durable long-term outcomes with low aorta-related mortality, although overall survival decreases with increasing preoperative risk.

**Perspective**

With expanding endovascular options for aortic arch aneurysms, selecting the optimal treatment strategy remains challenging. Defining “high-risk” patients is difficult, making comprehensive risk assessment essential for individualized treatment decisions.

Open surgical repair, including total arch replacement (TAR), remains the standard treatment for aortic arch aneurysms.^1^^,^^2^ Thoracic endovascular aortic repair (TEVAR) is widely recognized as a less-invasive alternative, particularly for high-risk patients. Although short-term outcomes are comparable between open repair and hybrid TEVAR, complications such as stroke, spinal cord injury, and reintervention are reported more frequently after hybrid TEVAR.^3^, ^4^, ^5^ More recently, branched endografts and totally endovascular techniques have been developed; however, their long-term durability remains uncertain.^6^, ^7^, ^8^, ^9^ Despite being more invasive than endovascular approaches, TAR remains a reliable and durable therapeutic approach, particularly when prioritizing long-term outcomes.^4^^,^^10^^,^^11^ In the era of evolving endovascular technologies, selecting appropriate patients remains a major challenge in managing aortic arch aneurysms. This study evaluated 25-year outcomes and established risk stratification in patients who underwent TAR with a 4-branched graft for nondissecting aortic arch aneurysms at Kobe University.

## Methods

### Ethics

This retrospective, single-center study was approved by the institutional review board of Kobe University School of Medicine (institutional review board no. B220178, March 13, 2025), and the requirement for written informed consent was waived.

### Study Design and Definitions

**Figure 1:**
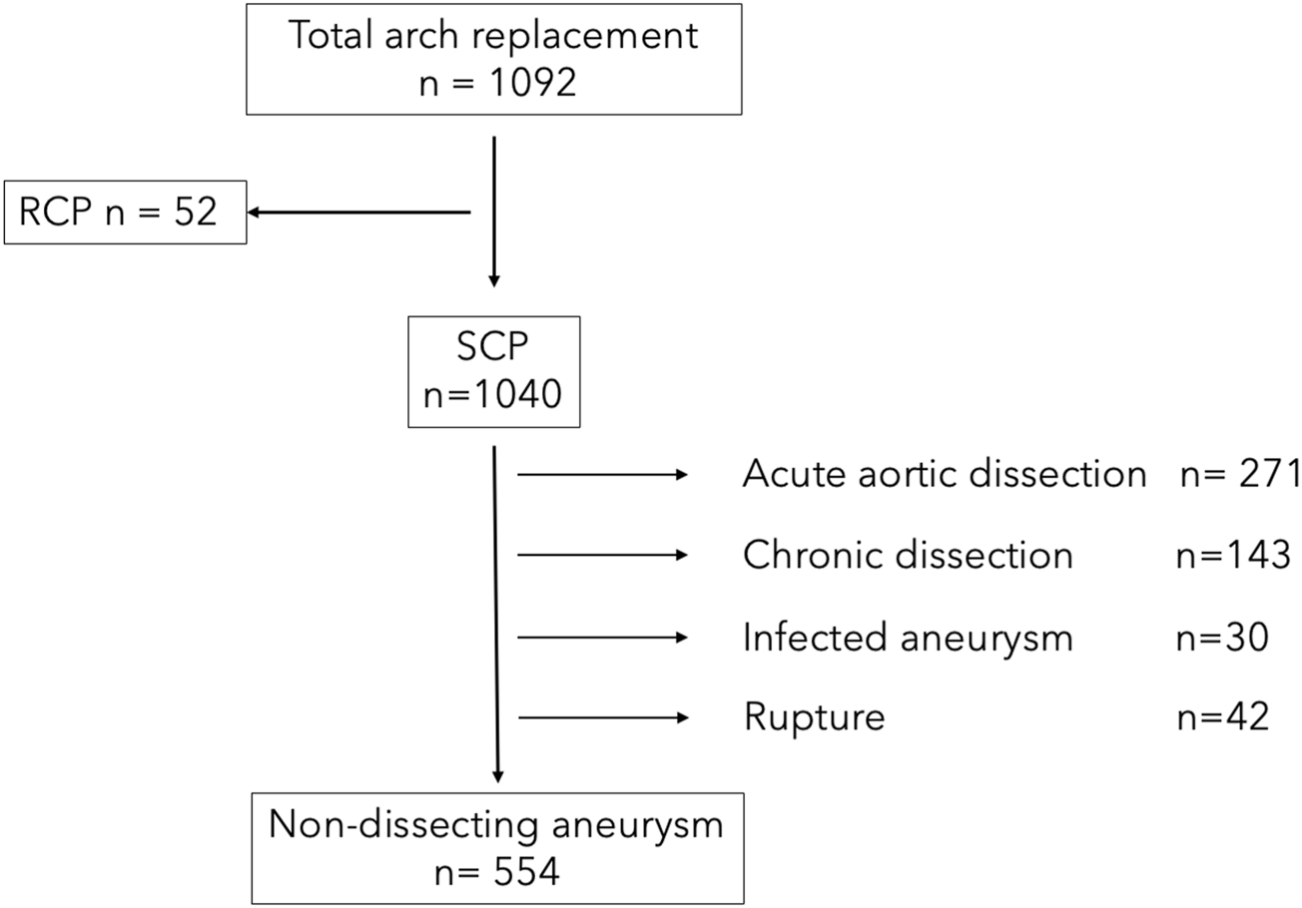
From October 1999 to December 2024, 1092 patients underwent TAR. After excluding patients with RCP, acute aortic dissection, chronic dissection, infected aneurysm, and rupture, 554 patients with nondissecting aortic arch aneurysms were included in this study. *TAR*, Total arch replacement; *RCP*, retrograde cerebral perfusion; *SCP*, selective cerebral perfusion.

From October 1999 to December 2024, 1092 patients underwent TAR. Fifty-two patients with retrograde cerebral perfusion were excluded, leaving 1040 patients who underwent TAR with selective cerebral perfusion (SCP). Fifty-two patients who underwent retrograde cerebral perfusion were intentionally excluded because these procedures were performed during the earliest period of our institutional experience, before SCP became our standard cerebral-protection strategy. This exclusion was made to avoid heterogeneity in cerebral-protection methods and to evaluate outcomes after TAR using a consistent contemporary SCP-based approach. Thirty patients with aortic infection and 42 with rupture were further excluded, resulting in 554 patients with nondissecting aortic arch aneurysms (Figure 1). All patients underwent elective surgery. Patients with acute or chronic aortic dissection were excluded because their clinical background and etiology differ from those of patients with nondissecting aortic arch aneurysms. In this study, nondissecting aortic arch aneurysm was defined as an aneurysmal disease involving the aortic arch requiring TAR, with the main aneurysmal pathology located within Ishimaru zones 0 to 3. Patients with aneurysmal disease extending beyond zone 4 were generally considered to have extensive thoracic aortic disease and were treated using either a staged strategy of TAR followed by TEVAR or a one-stage repair when appropriate. The primary end point was long-term overall survival. Secondary end points included 30-day mortality, hospital mortality, freedom from aorta-related death, and freedom from aortic events. Aorta-related death comprised both aortic and hospital deaths, whereas aortic events included aorta-related death or additional reinterventions. Subgroup analyses stratified patients by age, renal function, their combination, and the number of preoperative risk factors for late mortality. Patients were stratified into 4 age groups on the basis of distribution and clinical relevance: (1) ≤70, (2)71-79, (3) 80-84, and (4) ≥85 years. Renal function was assessed using estimated glomerular filtration rate (eGFR) and categorized according to chronic kidney disease stages: Ⅰ ≥60 mL/min/1.73 m^2^ (stage 1-2), Ⅱ 45-59 (stage 3a), Ⅲ 30-44 (stage 3b), and Ⅳ <30 (stage 4-5). Overall survival was compared across these groups. On the basis of survival patterns, age ≥85 years and eGFR <45 mL/min/1.73 m^2^ were identified as clinically relevant thresholds and included in the multivariable Cox proportional hazards model. Patients were further stratified into 4 groups on the basis of these cutoffs to evaluate their combined impact on long-term survival: group A, age <85 years and eGFR ≥45; group B, age <85 years and eGFR <45; group C, age ≥85 years and eGFR ≥45; and group D, age ≥85 years and eGFR <45. A multivariate Cox proportional hazards model was then used to identify independent preoperative predictors of late mortality. On the basis of these predictors, an exploratory risk-stratification model was developed according to the number of significant preoperative risk factors present (group a: 0; group b: 1; group c: 2; group d: ≥3), and survival was compared among groups. In an additional exploratory analysis, patients with available Japan surgical risk score (Japan SCORE) data were stratified according to a Japan SCORE cutoff of 7.62, corresponding to the upper quartile, and hospital mortality and overall survival were compared between groups. Patients were routinely followed once or twice per year at our outpatient clinic in collaboration with their primary physicians or referring hospital. Survival status and causes of death were obtained from medical records, referring or primary physicians, and family members. Causes of death were adjudicated on the basis of available clinical information. Sudden unexplained deaths were considered nonaorta-related unless an aorta-related event was clinically suspected or confirmed. Prolonged postoperative stay was defined as a postoperative hospital stay of 30 days or longer.

### Surgical Approach

Our surgical strategy for TAR using a 4-branched graft via median sternotomy has evolved over time.^12^^,^^13^ The arterial cannulation site was selected on the basis of preoperative imaging and intraoperative epiaortic echocardiography, particularly the distribution of atheromatous disease and calcification. In patients with severe atheromatous disease or shaggy aorta, cannulation and perfusion strategies were modified. When direct ascending aortic cannulation was considered feasible, a dispersive-tip aortic cannula (Stealth Flow; Senko Medical Instrument Mfg Co, Ltd) was used and directed toward the aortic valve. In selected patients, the subclavian artery was used instead of direct ascending aortic or arch cannulation. After the establishment of cardiopulmonary bypass, patients were cooled, and antegrade SCP was initiated during circulatory arrest. SCP was routinely performed through the bilateral carotid arteries and the left subclavian artery. Since 2002, antegrade SCP has been used for cerebral protection, with tympanic and rectal temperatures maintained at 23 °C and 30 °C, respectively. The distal aortic aneurysm is circumferentially transected beyond the left subclavian artery from inside the aorta, avoiding a longitudinal incision to prevent injury to the left recurrent laryngeal nerve. After distal anastomosis using the open distal anastomosis technique, lower body reperfusion and rewarming are initiated. The proximal anastomosis is then completed, followed by coronary reperfusion. Finally, all supra-aortic branches are reconstructed individually using a 4-branched graft, starting from the left subclavian artery and proceeding to the brachiocephalic artery. When an elephant trunk was not used, we generally created a landing zone of at least 2 cm between the left subclavian artery branch and the distal anastomosis.

For patients with extensive aortic arch aneurysm, a staged strategy of TAR followed by TEVAR has been adopted. When a staged approach is not feasible, a one-stage procedure is considered in patients who are operable.^14^^,^^15^ The anterolateral thoracotomy with a partial sternotomy approach has been used as a representative surgical option for extensive aortic arch aneurysms. In summary, a skin incision is made from the bottom of the xiphoid to the anterior axillary line in a convex curved fashion, and the thorax is entered through the third intercostal space with a partial lower sternotomy. Cardiopulmonary bypass is established in a manner very similar to TAR through median sternotomy. In contrast to the standard approach, femoral artery cannulation is additionally performed before opening the aortic arch, and the descending aorta is clamped to maintain lower-body perfusion. Frozen elephant trunk (FET) was not routinely used for nondissecting aneurysms at our institution.

### Statistical Analysis

All analyses were performed using JMP for Macintosh, version 19.0.4 (SAS Institute). Continuous variables are presented as mean ± standard error and were compared using Student *t* test or analysis of variance, as appropriate. Categorical variables were compared using the χ^2^ or Fisher exact test. Overall survival, freedom from aorta-related death, and freedom from aortic events were analyzed using the Kaplan-Meier method and compared with the log-rank test. Age, renal function (eGFR), combined age-renal function categories, and the number of preoperative risk factors were analyzed as categorical variables, with the lowest-risk category serving as the reference group. For, age, renal function, combined age-renal function, and the number of preoperative risk factors, pairwise Cox proportional hazards analyses were additionally performed to compare hazard ratios between categories. Hazard ratios with 95% CIs were calculated. Variables with *P* < .05 in univariate analysis were included in a multivariate Cox proportional hazards model to identify independent predictors of late mortality. The proportional hazards assumption was assessed graphically using log-minus-log survival plots. Following the methods of Finkelstein and colleagues,^16^ we compared patient survival with the age- and sex-matched Japanese general population using the life table published by the Ministry of Health, Labour and Welfare.^17^

## Results

### Patients’ Characteristics and Operative Details

From October 1999 to December 2024, 554 patients with nondissecting aortic arch aneurysms underwent TAR. Preoperative characteristics are summarized in Table 1. The mean age was 73.6 ± 9.9 years, and the mean Japan SCORE was 6.6 ± 5.9. Elephant trunk and FET were used in 109 (19.6%) and 14 (2.5%) patients, respectively. Concomitant coronary artery bypass grafting (CABG) and root replacement were performed in 155 (27.9%) and 17 (3.0%) patients, respectively. The majority (534, 96.3%) underwent TAR via median sternotomy. A one-stage repair strategy was used in 20 patients (3.6%). Operative times are presented in Table 2.

**Table 1:** Preoperative characteristics

Variables	Overall (n = 554)	Group 1 (n = 132)	Group 2 (n = 276)	Group 3 (n = 112)	Group 4 (n = 34)	*P* value
Japan SCORE	6.6 ± 5.9	4.0 ± 0.5	6.9 ± 0.3	7.3 ± 0.5	9.5 ± 0.9	<.0001
Age, y	73.6 ± 9.9	60.6 ± 0.5	74.8 ± 0.3	81.7 ± 0.5	86.8 ± 1.0	<.0001
Male	429 (77.4)	107 (81.6)	219 (79.3)	78 (69.6)	25 (73.5)	.652
BMI	23.5 ± 3.3	23.8 ± 0.2	23.4 ± 0.1	23.4 ± 0.3	23.1 ± 0.5	.136
Marfan syndrome	7 (1.2)	7 (5.3)	0 (0.0)	0 (0.0)	0 (0.0)	.0001
HT	373 (67.3)	83 (62.8)	189 (68.4)	75 (66.9)	26 (76.4)	.439
DL	207 (37.3)	41 (31.0)	104 (37.6)	50 (44.6)	12 (35.2)	.182
DM	108 (19.4)	24 (18.1)	60 (21.7)	17 (15.1)	7 (20.5)	.484
Shaggy	57 (10.2)	11 (8.3)	29 (10.5)	11 (9.8)	6 (17.6)	.511
Smoking	302 (54.5)	70 (53.0)	153 (55.4)	60 (53.5)	19 (55.8)	.964
COPD	98 (17.6)	12 (9.0)	55 (19.9)	22 (19.6)	9 (26.4)	.013
History of CVD/PAD	139 (25.0)	31 (23.4)	75 (27.1)	23 (20.5)	10 (29.4)	.488
Low EF <40%	12 (2.1)	2 (1.5)	5 (1.8)	3 (2.6)	2 (5.8)	.549
Re-sternotomy	20 (3.6)	0 (0.0)	12 (4.3)	5 (4.4)	3 (8.8)	.006
Previous valve surgery	2 (0.3)	1 (0.7)	1 (0.3)	0 (0.0)	0 (0.0)	.683
Previous CABG or PCI	41 (7.4)	13 (9.8)	17 (6.1)	8 (7.1)	0 (0.0)	.081
Previous aortic surgery	38 (6.8)	9 (6.8)	22 (7.9)	7 (6.2)	3 (8.3)	.915
Elephant or FET	123 (22.2)	24 (18.1)	65 (23.5)	26 (23.2)	8 (23.5)	.641
Follow-up length, y	5.6 ± 4.2	7.6 ± 4.5	5.4 ± 4.0	4.3 ± 3.7	3.8 ± 3.6	<.0001

*Japan SCORE*, Japan surgical risk score; *BMI*, body mass index; *HT*, hypertension; *DL*, dyslipidemia; *DM*, diabetes mellitus; *COPD*, chronic obstructive pulmonary disease; *CVD*, cerebrovascular disease; *PAD*, peripheral artery disease; *EF*, ejection fraction; *CABG*, coronary artery bypass grafting; *PCI*, percutaneous coronary intervention; *FET*, frozen elephant trunk.

**Table 2:** Operative time

Variable	Mean ± SD
Operation time, min	401.4 ± 127.0
ECC time, min	190.6 ± 62.7
ACC time, min	87.7 ± 47.4
CA (lower body) time, min	43.1 ± 19.2
SCP time, min	105.4 ± 37.6

*ECC*, Extracorporeal circulation; *ACC*, aortic crossclamp; *CA*, circulatory arrest; *SCP*, selective cerebral perfusion.

### Early Outcomes

The 30-day mortality was 1.0% (n = 6), caused by perioperative myocardial infarction (n = 1), multiple organ failure (n = 1), and intestinal necrosis (n = 3). Hospital mortality was 3.6% (n = 20). Excluding 30-day deaths, the remaining 14 hospital deaths were attributable to pneumonia (n = 9), pancreatitis (n = 2), intestinal necrosis (n = 2), and disseminated intravascular coagulation (n = 1). Temporary neurologic dysfunction, permanent neurologic dysfunction, and spinal cord injury occurred in 27 (4.8%), 13 (2.3%), and 7 (1.2%) patients, respectively. Postoperative hoarseness occurred in 33 patients (5.9%), and tracheostomy was required in 35 (6.3%).

### Long-Term Outcomes

**Figure 2:**
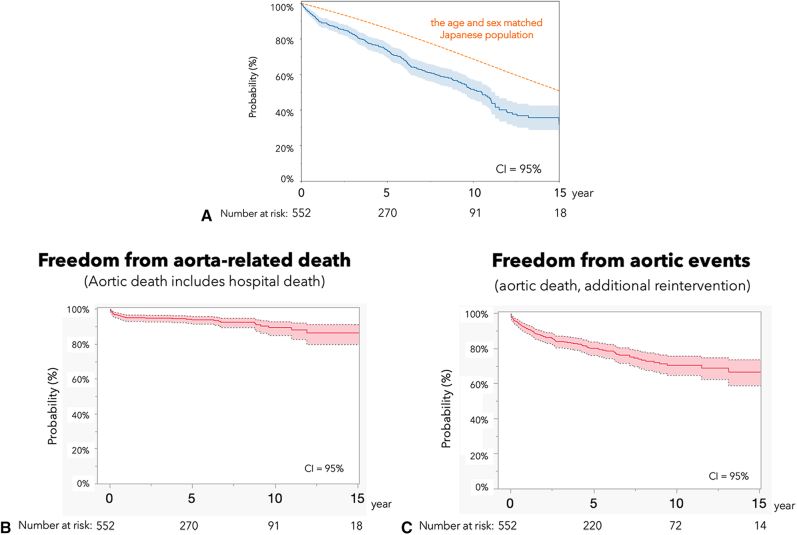
Kaplan-Meier curves with 95% CIs. A, Overall survival in the entire cohort (*blue line*) and overall survival compared with age- and sex-matched Japanese population (*orange dotted line*). B, Freedom from aorta-related death. C, Freedom from aortic events. *RCP*, Retrograde cerebral perfusion; *SCP*, selective cerebral perfusion.

Overall survival was 51.8 ± 2.8% at 10 years, compared with 68.6% in the age- and sex-matched Japanese population (Figure 2, *A*). At 10 years, freedom from aorta-related death (Figure 2, *B*) and freedom from aortic events (Figure 2, *C*) were 89.5 ± 1.9% and 70.5 ± 2.8%, respectively. Causes of aorta-related death, excluding hospital deaths, included aortic rupture (n = 13; abdominal aorta, n = 6; descending aorta, n = 5; thoracoabdominal aorta, n = 2), graft infection (n = 2), aortoesophageal fistula (n = 2), pneumonia associated with hoarseness (n = 1), and iliac limb occlusion after endovascular aortic repair (n = 1). The most common additional reintervention was TEVAR (n = 35), followed by endovascular aortic repair (n = 21). Among the 35 patients who required TEVAR during follow-up, one underwent TEVAR for a pseudoaneurysm at the distal anastomosis, one underwent zone 2 TEVAR, and 33 underwent TEVAR for descending thoracic aortic aneurysms. Among these 33 patients, 22 had undergone TAR with an elephant trunk and 2 had undergone TAR with FET trunk at the initial operation.

### Subgroup Analyses

**Figure 3:**
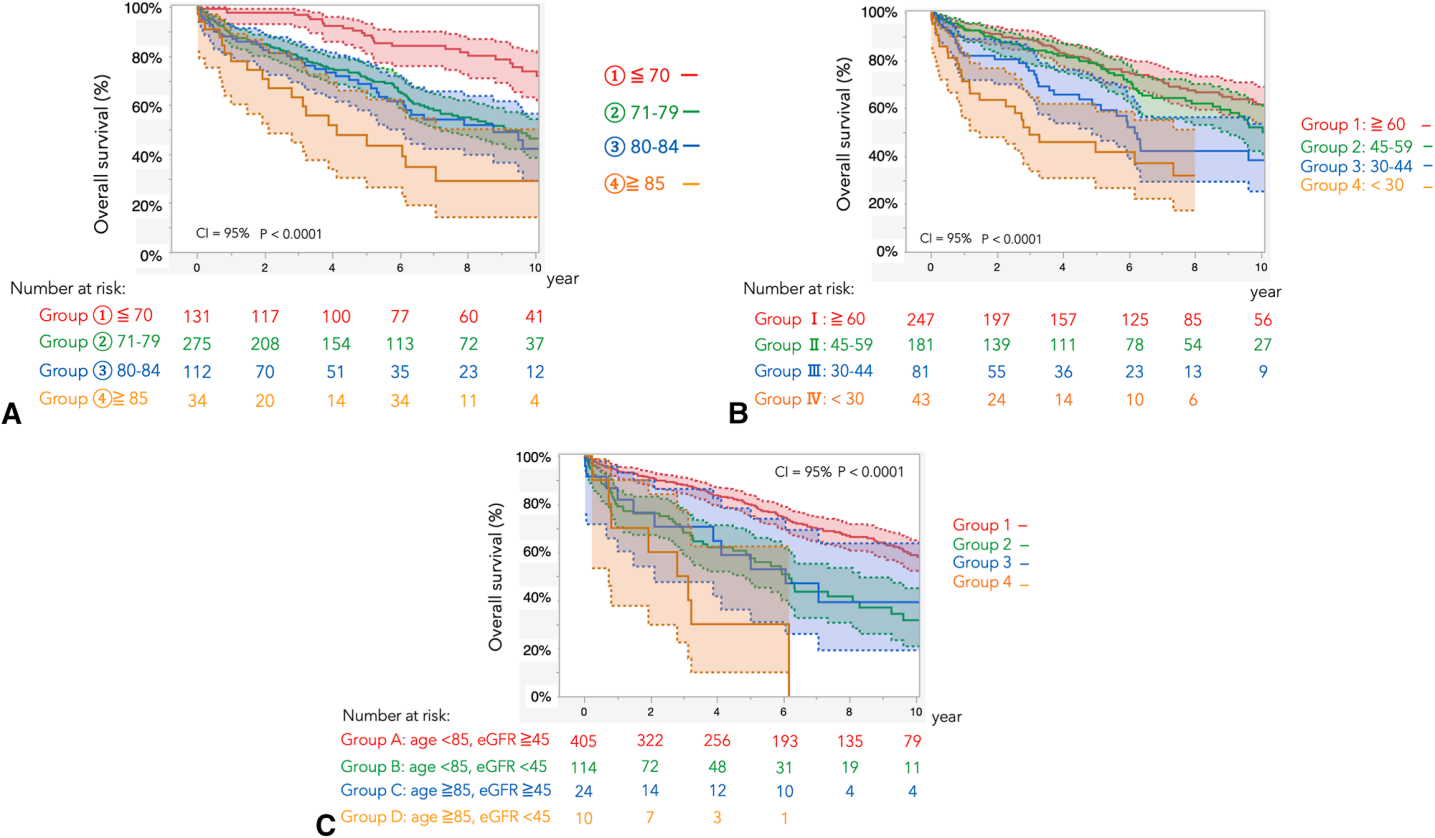
Kaplan-Meier curves for overall survival with 95% CIs. A, Age stratification. B, Renal function stratification. C, Combined age and renal function.

**Figure E1:**
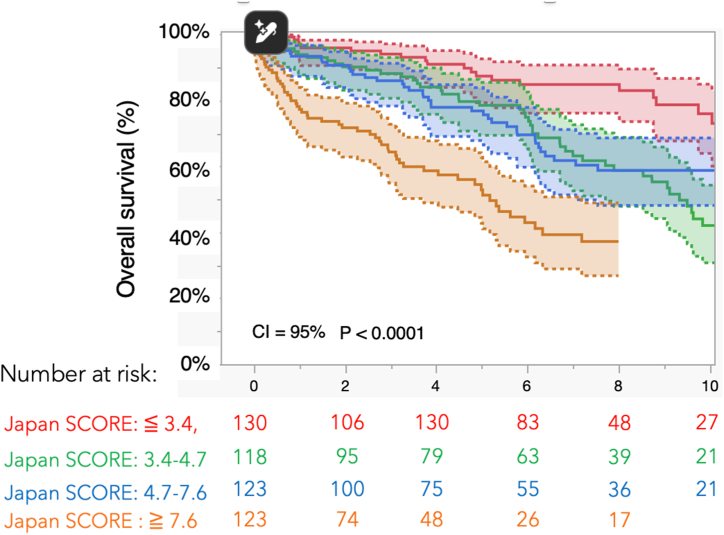
Kaplan-Meier curves for overall survival with 95% CIs. Japan SCORE stratification. *Japan SCORE*, Japan surgical risk score.

**Figure E2:**
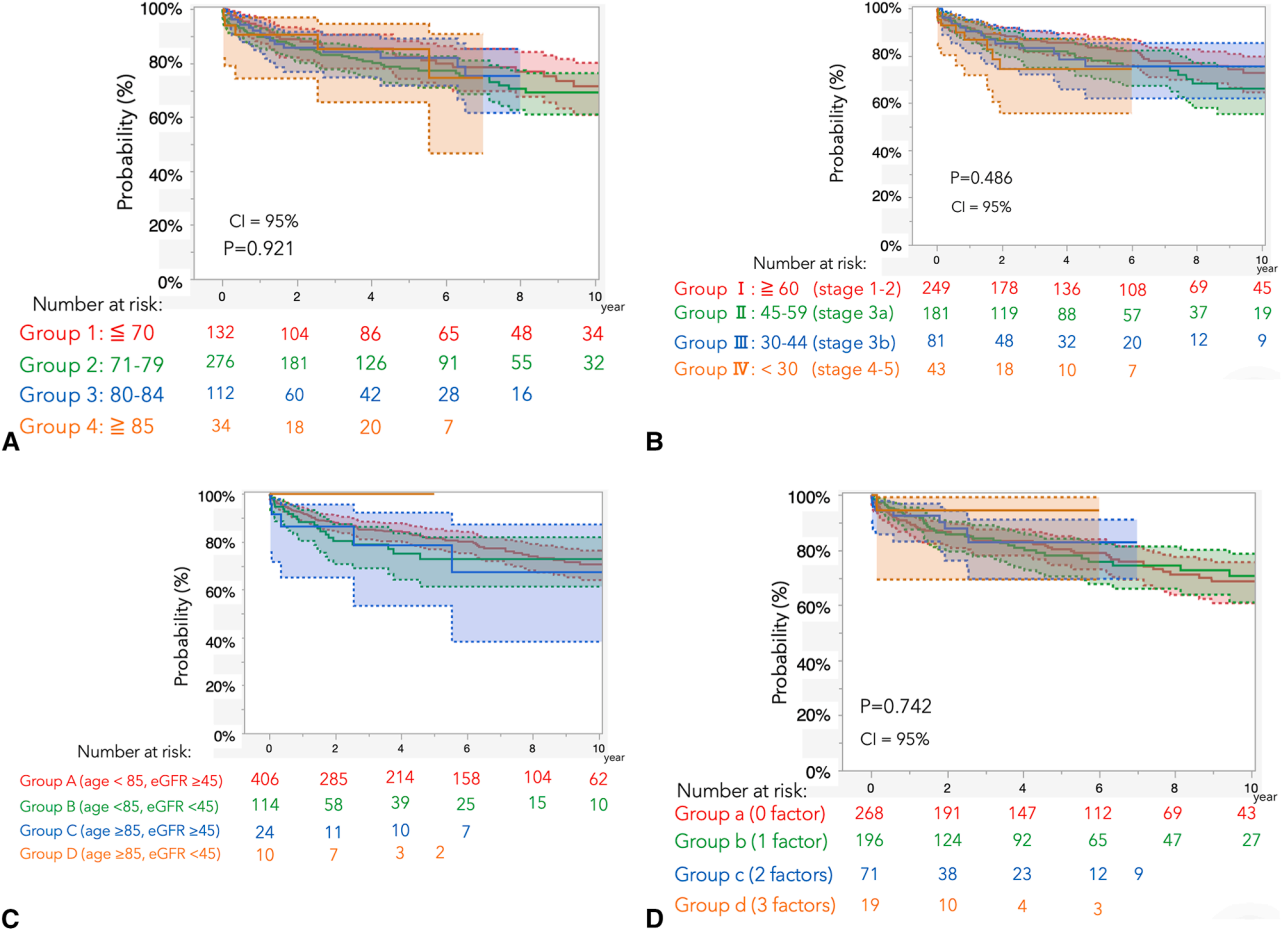
Kaplan-Meier curves for freedom from aortic events with 95% CIs. A, Age stratification. B, Renal function stratification. C, Combined age and renal function. D, Stratification by number of preoperative risk factors. *eGFR*, Estimated glomerular filtration rate.

**Figure E3:**
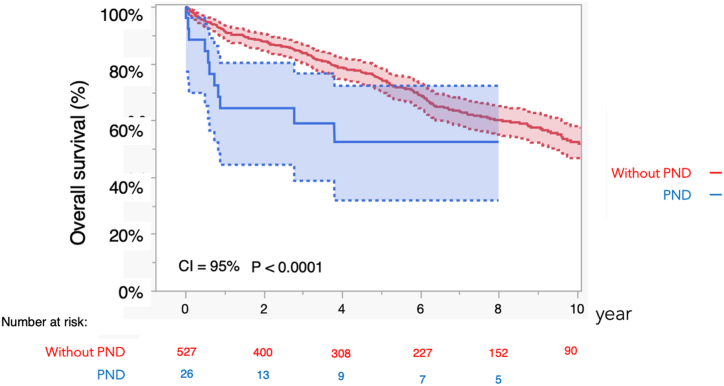
Kaplan-Meier curves for overall survival with 95% CIs. Permanent neurologic dysfunction (34.9 ± 16.0% at 10 years) was associated with significantly worse overall survival than those without PND (52.3 ± 2.8% at 10 years). (*P* = .013). *PND*, Permanent neurologic dysfunction.

**Figure E4:**
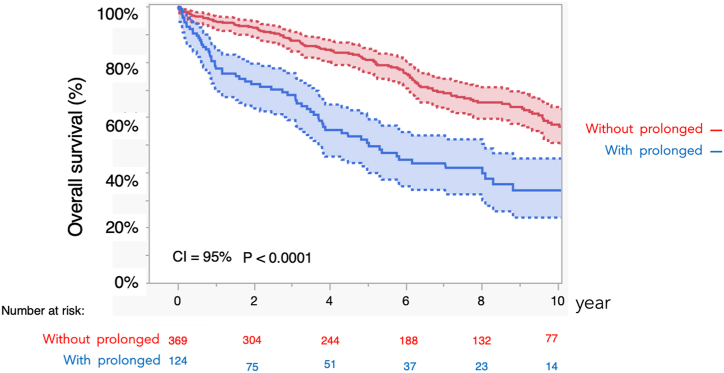
Kaplan-Meier curves for overall survival with 95% CIs. Patients with prolonged postoperative hospital stay (33.4 ± 5.5% at 10 years) had significantly worse overall survival than those without prolonged postoperative stay (57.2 ± 3.3% at 10 years) (*P* < .0001).

For age stratification (group 1, ≤70 years; group 2, 71-79; group 3, 80-84; group 4, ≥85; Table 1), overall survival differed significantly among groups (*P* < .0001, Figure 3, *A*), with 10-year survival rates of 74.3 ± 4.8%, 46.3 ± 4.0%, 42.2 ± 7.1%, and 28.9 ± 9.4% for groups 1 to 4, respectively. In Cox analysis, age ≥85 years was associated with markedly worse overall survival compared with younger groups. For renal function stratification (group I eGFR ≥60; group II, 45-59; group III, 30-44; group IV, <30), 10-year overall survival rates were 61.8 ± 3.9% (n = 249), 52.0 ± 5.0% (n = 181), 38.3 ± 7.4% (n = 81), and 14.1 ± 8.1% (n = 43), respectively (*P* < .0001, Figure 3, *B*). Patients with eGFR ≥45 mL/min/1.73 m^2^ (groups I and II) had significantly better overall survival than those with eGFR <45 (groups III and IV). For combined age and renal function stratification (group A, age <85, eGFR ≥45; group B, age <85, eGFR <45; group C age ≥85, eGFR ≥45; group D, age ≥85, eGFR <45), 10-year overall survival rates were 58.8 ± 3.2% (n = 406), 31.6 ± 6.2% (n = 114), 39.1 ± 12.1% (n = 24), and 0.0% (n = 10) for groups A-D, respectively (*P* < .0001, Figure 3, *C*). Patients in group D, with both advanced age and impaired renal function, had the worst survival. Among patients with available Japan SCORE data, those with a Japan SCORE ≥7.6 had a hospital mortality of 11.4% (Table E1), numerically exceeding the score threshold, and a 10-year overall survival of 26.3% ± 6.1% (Figure E1). No significant differences in 10-year freedom from aortic events were observed according to age, renal function, or the combined stratification of age and renal function (Figure E2, *A*-*C*). In additional analyses, permanent neurologic dysfunction was associated with significantly worse overall survival (Figure E3). Similarly, patients with prolonged postoperative hospital stay had significantly worse overall survival than those without prolonged postoperative stay (Figure E4).

### Risk Analysis for Late Death

**Figure 4:**
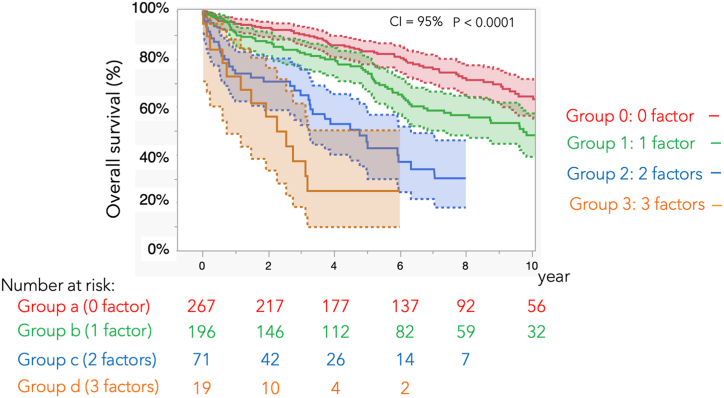
Kaplan-Meier curves for overall survival with 95% CIs. Stratification by number of preoperative risk factors.

Multivariable analysis identified age ≥85 years, eGFR <45 mL/min/1.73 m^2^, chronic obstructive pulmonary disease (COPD), history of cerebrovascular disease (CVD) and peripheral artery disease (PAD), and longer extracorporeal circulation (ECC) time as independent risk factors for late mortality (Table 3). In an additional analysis limited to patients aged <80 years, eGFR <45 mL/min/1.73 m^2^, COPD, history of CVD/PAD, and one-stage extensive repair were independently associated with late mortality (Table E2). Of these, age ≥85 years, eGFR <45, COPD, and history of CVD and PAD were preoperative factors, whereas longer ECC time was an operative factor. Risk stratification was performed according to the number of preoperative risk factors (0, 1, 2, or ≥3). A total of 298 patients had 0 risk factors (group a), 225 had 1 (group b), 82 had 2 (group c), and 21 had ≥3 (group d). Ten-year overall survival decreased progressively with increasing preoperative risk factors: 64.5 ± 3.9% in group a, 48.1 ± 4.6% in group b, 20.1 ± 7.5% in group c, and 12.4 ± 10.2% in group d (*P* < .0001, Figure 4; Table E3). There were no significant differences in 10-year freedom from aortic events among 4 groups (Figure E2, *D*).

**Table 3:** Risk factors for late mortality

Variable	Univariate HR (95% CI)	*P* value	Multivariate HR (95% CI)	*P* value
Age ≥85 y	2.50 (1.59-3.93)	<.001	2.32 (1.45-3.69)	<.001
eGFR <45	2.39 (1.78-3.21)	<.001	2.10 (1.55-2.83)	<.001
Male	1.29 (0.91-1.84)	.146	–	–
HT	1.08 (0.80-1.47)	.584	–	–
DM	1.10 (0.79-1.55)	.551	–	–
COPD	1.93 (1.38-2.68)	<.001	1.99 (1.42-2.79)	<.001
History of CVD/PAD	1.83 (1.37-2.43)	<.001	1.66 (1.23-2.22)	<.001
ECC (per unit)	1.003 (1.001-1.015)	.001	1.003 (1.000-1.005)	.011
Extensive (one stage)	1.93 (0.94-3.92)	.069	–	–
Shaggy	1.13 (0.71-1.80)	.585	–	–
CABG	1.75 (1.33-2.31)	<.001	1.25 (0.93-1.68)	.126
Root replacement	0.53 (0.22-1.31)	.173	–	–
Elephant or FET	0.91 (0.63-1.31)	.634		

*HR*, Hazard ratio; *eGFR*, estimated glomerular filtration rate; *HT*, hypertension; *DM*, diabetes mellitus; *COPD*, chronic obstructive pulmonary disease; *CVD*, cardiovascular disease; *PAD*, peripheral artery disease; *ECC*, extracorporeal circulation time; *CABG*, coronary artery bypass grafting; *FET*, frozen elephant trunk.

## Discussion

In this study of 554 patients with nondissecting aortic aneurysms undergoing TAR with SCP, the 10-year overall survival was 51.8 ± 2.8% compared with 68.6% in the age- and sex-matched Japanese population. Notably, 10-year freedom from aorta-related death remained high at 89.5 ± 1.9%. Stratified analyses showed that patients with both advanced age and impaired renal function had the poorest survival. In addition, long-term survival declined progressively with an increasing number of preoperative risk factors for late mortality.

Compared with the age- and sex-matched Japanese population, overall survival was lower, likely reflecting the relatively high preoperative risk of our cohort, as indicated by a mean Japan SCORE of 6.6 ± 5.9. Freedom from aorta-related death remained favorable, indicating that TAR effectively prevents aortic death and provides long-term benefit, even in high-risk patients. In contrast, recent guidelines highlight an increasing role for endovascular approaches in aortic arch aneurysm repair.^2^ Commercially available thoracic stent grafts were introduced in Japan in 2008. During the period in which TEVAR was available at our institution, 91 patients underwent TEVAR or hybrid endovascular repair for aortic arch pathology (zone 0-3) at our institution. These patients had relatively high preoperative risk profiles, with a mean age of 79.3 ± 8.5 years, Japan SCORE of 15.0 ± 14.3, and frailty scale of 4.5 ± 1.1. Thus, endovascular therapy was considered for selected high-risk patients. However, greater-risk patients were not systematically directed to endovascular therapy solely on the basis of age or comorbidity. Although endovascular approaches may be preferred for high-risk patients, the definition of “high risk” varies across institutions, and standardized criteria remain lacking.

For age stratification, patients were divided into 4 groups: group 1 (≤70 years), group 2 (71-79), group 3 (80-84), and group 4 (≥85 years). In our cohort, the first quartile, median, and third quartile were 70, 76, and 80 years, respectively. Clinically, grouping all patients older than 80 years together does not reflect what we observe in practice. Therefore, patients classified as octogenarian were divided into 2 groups. Patients aged ≥85 years had significantly worse survival compared with those aged 80 to 84 years, whereas no significant difference was observed between patients aged 71 to 79 years and 80 to 84 years. These findings support the validity of our age-based stratification. For renal function (group Ⅰ eGFR ≥60; group Ⅱ, 45-59; group Ⅲ, 30-44; group Ⅳ, <30), patients with eGFR ≥45 (groups Ⅰ and Ⅱ) had significantly better overall survival than those with eGFR <45 (groups Ⅲ and Ⅳ). On the basis of these analyses, cutoff values of age ≥85 and eGFR <45 were used to create combined stratification groups: group 1 (age <85 years, eGFR ≥45), group 2 (age <85 years, eGFR <45), group 3 (age ≥85 years, eGFR ≥45), and group 4 (age ≥85 years, eGFR <45). Patients in group D (age ≥85 years, eGFR <45) had the worst survival, although no significant difference was observed between groups B and C. Furthermore, overall survival decreased significantly as the number of independent preoperative risk factors increased. These results suggest that “high risk” is determined not by a single factor but by the accumulation of multiple factors. Accordingly, the optimal strategy should be tailored using a patient-centered approach on the basis of individual characteristics. In contrast, freedom from aortic events did not differ significantly among the groups. This finding suggests that patient background factors had a stronger impact on overall survival than on late aortic events.

Multivariable analysis also identified longer ECC time as an independent risk factor for late mortality, suggesting that minimizing ECC time may improve long-term outcomes. In our strategy, complete resection of the aortic aneurysm remains essential for nondissecting aneurysms. Spinal cord injury is a recognized postoperative complication^18^^,^^19^; therefore, FET is generally avoided at our institution. Nevertheless, FET may be considered in selected cases to reduce surgical invasiveness.

Shaggy aorta is recognized particularly in East Asian countries as an important risk factor in aortic surgery.^20^^,^^21^ Contrary to expectations, it was not an independent risk factor for late mortality in this study, likely reflecting careful preoperative assessment using detailed imaging. In affected patients, we modify the surgical strategy with meticulous cannulation,^22^ antegrade perfusion,^23^ brain-isolation techniques,^24^ and nonclamping approaches.^22^^,^^25^ These tailored strategies may have mitigated the associated risk. Concomitant CABG was associated with worse unadjusted survival but was not an independent risk factor for late mortality after adjustment, consistent with our previous report.^26^ This may reflect the fact that worse outcomes in patients requiring CABG are more related to the underlying burden of coronary artery disease and systemic atherosclerosis than to the CABG procedure itself. In addition, concomitant CABG may have reduced perioperative myocardial ischemia and subsequent coronary events.

Previous studies of hybrid TEVAR and emerging branched endovascular techniques for aortic arch aneurysms have reported acceptable early outcomes, particularly in elderly or high-risk patients for whom less-invasive treatment is desirable. However, concerns remain regarding neurologic complications, endoleak, reintervention, and uncertain long-term durability.^5^, ^6^, ^7^, ^8^ In contrast, TAR is more invasive and requires cardiopulmonary bypass, circulatory arrest, and cerebral protection but provides definitive aneurysm exclusion and durable aorta-related outcomes.^4^^,^^9^^,^^10^ The present results may serve as a useful benchmark for contemporary TAR performed at an experienced open aortic center. However, the generalizability of these outcomes should be interpreted with caution, because results after TAR are influenced by institutional volume, surgical expertise, standardized operative strategy, and perioperative management. We believe that favorable outcomes may be achievable in other centers when TAR is performed using a consistent strategy and appropriate patient selection. Nevertheless, each institution should select the treatment approach best suited to its expertise, including TAR or endovascular repair. Rather than defining TEVAR and TAR as competing strategies, these approaches should be considered complementary options. In elderly patients, particularly those aged ≥80 or ≥85 years, the decision to proceed with TAR was determined by functional status, frailty, cognitive function, cardiac function, pulmonary reserve, renal function, comorbidities, anatomy, anticipated life expectancy, rupture risk, and patient preference. TAR was generally offered only when the patient was considered likely to tolerate surgery and to derive meaningful long-term benefit. Therefore, the present cohort reflects carefully selected patients, particularly among the very elderly population.

### Limitations

This study has several limitations. First, it is a retrospective, nonrandomized, single-center study. Although the overall cohort was relatively large, certain subgroups were small, particularly patients aged ≥85 years, those with eGFR <30 mL/min/1.73 m^2^, and those with multiple preoperative risk factors (3 risk factors). Consequently, statistical power in these subgroups may have been limited. Second, detailed anatomic, morphologic, and pathologic classifications were not consistently available throughout the 25-year study period, including aneurysm dimensions, complete Ishimaru zone classification, giant cell aortitis, bicuspid aortic valve-associated aortopathy, and saccular versus mega-aorta morphology. Although severe atheromatous disease was assessed clinically as shaggy aorta, more granular classification was not incorporated into the analysis. Third, frailty data were not included, because scores were not consistently available and no universally accepted frailty scale exists for patients undergoing aortic surgery. In clinical practice, surgical decision-making often relies on a comprehensive frailty assessment that combines objective evaluation with clinical judgment. Future studies should incorporate frailty into risk assessment models.

Fourth, because this study included only patients who underwent TAR at a high-volume open aortic center, treatment selection was influenced by institutional expertise and surgical preference. Selection bias is therefore unavoidable, and the results should not be interpreted as directly comparable with outcomes after endovascular or hybrid repair. Finally, long-term patency of the reconstructed cervical branches, including the left subclavian artery branch, was not systematically quantified as a predefined end point. Although contrast-enhanced computed tomography was routinely performed when renal function allowed and clinically significant branch occlusion requiring reintervention was extremely rare in our experience, standardized patency analysis was not performed in all patients.

## Conclusions

In this relatively high-risk cohort, TAR provided durable long-term outcomes with excellent freedom from aorta-related death. Overall survival declined with the accumulation of preoperative risk factors, emphasizing the importance of comprehensive risk assessment. Optimal treatment strategies should therefore be individualized through a patient-centered approach.

## Conflict of Interest Statement

The authors reported no conflicts of interest.

The *Journal* policy requires editors and reviewers to disclose conflicts of interest and to decline handling or reviewing manuscripts for which they may have a conflict of interest. The editors and reviewers of this article have no conflicts of interest.

## Figures and Tables

**Table E1:** Hospital mortality stratified by Japan SCORE

Japan SCORE	Hospital mortality
≤3.4 (n = 130)	0%
>3.4 to ≤4.7 (n = 118)	0.9%
>4.7 to <7.62 (n = 124)	3.2%
≥7.62 (n = 123)	11.4%

*Japan SCORE*, Japan surgical risk score.

**Table E2:** Risk factors for late mortality in patients aged <80 years

Variable	Univariate HR (95% CI)	*P* value	Multivariate HR (95% CI)	*P* value
eGFR <45	2.30 (1.62-3.28)	<.001	2.01 (1.40-2.90)	<.001
Male	1.40 (0.89-2.21)	.137	–	–
HT	1.08 (0.76-1.53)	.661	–	–
DM	1.22 (0.82-1.82)	.318	–	–
COPD	1.97 (1.31-2.96)	.001	2.34 (1.54-3.56)	<.001
History of CVD/PAD	1.89 (1.35-2.65)	<.001	1.73 (1.22-2.45)	.002
ECC (per unit)	1.003 (1.001-1.006)	.006	1.002 (0.999-1.005)	.135
Extensive (one stage)	2.29 (1.06-4.92)	.033	2.34 (1.06-5.15)	.034
Shaggy	1.23 (0.72-2.11)	.436	–	–
CABG	1.72 (1.23-2.40)	.001	1.41 (0.98-2.01)	.0057
Root replacement	0.49 (0.18-1.33)	.163	–	–
Elephant or FET	0.73 (0.48-1.11)	.145		

*HR*, Hazard ratio; *eGFR*, estimated glomerular filtration rate; *HT*, hypertension; *DM*, diabetes mellitus; *COPD*, chronic obstructive pulmonary disease; *CVD*, cardiovascular disease; *PAD*, peripheral artery disease; *ECC*, extracorporeal circulation time; *CABG*, coronary artery bypass grafting; *FET*, frozen elephant trunk.

**Table E3:** Cox proportional hazards analysis by number of preoperative risk factors

Cox model	HR (95% CI)	*P* value
b vs a	1.66 (1.21-2.27)	<.0001
c vs a	4.23 (2.87-6.24)	<.0001
d vs a	8.02 (4.46-14.4)	<.0001
c vs b	2.54 (1.73-3.73)	<.0001
d vs b	4.82 (2.69-8.63)	<.0001
d vs c	1.89 (1.02-3.48)	.040

*HR*, Hazard ratio.
